# The hunt for mammalian epialleles

**DOI:** 10.1186/1753-6561-6-S6-O25

**Published:** 2012-10-01

**Authors:** Vardhman Rakyan

**Affiliations:** 1 Barts and the London School of Medicine and Dentistry, London, UK

## 

Epialleles are genomic loci at which the epigenetic state can stably vary amongst individuals of a given population. Although first described and still best understood in plants, in recent years we have come to realize that epigenomic landscapes in mammals can also show considerable inter-individual variation. Such mammalian epialleles could arise through genetic influences, or have non-genetic origins as a result of stochastic events, environmental factors such as exposure to a compromised *in utero* environment, or adult lifestyle-associated factors such as smoking. My lab is currently pursuing several complementary lines of investigation that integrate molecular genetics and epigenomics in mouse models and human cohorts to understand the role of epialleles in complex phenotypes and diseases. In my talk, I will present a synthesis of the latest findings from several ongoing studies in my lab on epiallelic variation in mammals.

